# Editorial: Characterization of major traits and identification of functional genes for potato

**DOI:** 10.3389/fpls.2023.1204304

**Published:** 2023-05-26

**Authors:** Juan Du, Vivianne G. A. A. Vleeshouwers, Botao Song

**Affiliations:** ^1^ National Key Laboratory for Germplasm Innovation & Utilization of Horticultural Crops, Huazhong Agricultural University, Wuhan, China; ^2^ Key Laboratory of Potato Biology and Biotechnology, Ministry of Agriculture and Rural Affairs, Huazhong Agricultural University, Wuhan, China; ^3^ College of Life Science and Technology, Huazhong Agricultural University, Wuhan, China; ^4^ Plant Breeding, Wageningen University and Research, Wageningen, Netherlands

**Keywords:** potato, gene, development, yield, nutrition, abiotic/biotic, plant immunity, disease resistance

Potato, *Solanum tuberosum*, is the fourth largest food crop and the most important non-grain crop in the world, as well as the model plant for the development of abnormal organs. It originates from the mountains of the Andes, and a great diversity of related wild tuber-bearing *Solanum* species thrive in South, Central, and North America. The widely cultivated potato has wide adaptability, high yield, and comprehensive nutrition. It can be used as the main food as well as vegetable and feed. Potato cultivars are autotetraploid (2n = 4x = 48), with highly heterozygous chromosomes and complex inheritance. Besides, artificial selection during cultivation has resulted in a relatively narrow genetic base, low heterosis, poor biotic and abiotic resistance, and so on. Unclear mechanisms of major potato traits, including growth and development, tuber nutrition and morphology, tolerance to abiotic and resistance to the many diseases, are key bottlenecks restricting potato genetic improvement. The decoding of several cultivated and wild potato genomes coupled with rapid advances in bioinformatics has provided powerful tools for detailed genetic and functional analysis. This Research Topic showcases exciting findings in potato ranging from tuber development, potato yield, tuber quality, abiotic and biotic stress resistance.

Potato tubers provide starch, which is the largest source of carbohydrates in the human diet. Cell wall invertase (CWI) is an essential coordinator in carbohydrate partitioning and sink strength determination, thereby playing key roles in plant development. Liu et al. identified StInvInh1 as a key inhibitor of CWI and demonstrated that its inhibition leads to improved microtuber development and higher accumulations of dry matter. These findings provide new insights into the regulation of tuber development. In addition to nutrient composition, tuber shape is one of the most important traits for potato breeding. To efficiently identify QTL for tuber shape, Huang et al. performed QTL-Seq analysis and linkage analysis in a diploid potato population-PM7. They identified a major stable QTL *TScha6* in a 1.85 Mb interval on chr 6 for tuber shape, and subsequently verified the QTL in a natural mapping population. Potato tuber sprouting has a great impact on processing and storage, but the molecular mechanism of sprouting is unclear. Wang et al. found that overexpression of *StTCP15* leads to early sprouting of potato tubers, while down-regulated expression of the *StTCP15* gene leads to the dormancy of potato tubers. Through further research, they suggested that *StTCP15* regulated potato tuber dormancy and sprouting by affecting the dynamic balance between ABA and GA3. Altogether, these studies provide insight in the starch content, size, shape and sprouting of potato tubers.

The major threat to the sustainable production of potatoes remains the pressure of many diseases and pests. The most devastating disease is late blight caused by the oomycete *Phytophthora infestans*. Breeding for resistance to late blight has so far mainly been focused on the introgression of resistance genes, which are typically quickly defeated by this pathogen. Pattern-triggered immunity (PTI) mediated by plant pattern recognition receptors (PRRs) provides a promising alternative as they recognize conserved MAMPs, leading to enhanced broad-spectrum disease resistance. To study the mechanisms of PTI against *P. infestans*, Mu et al. performed a sequential windowed acquisition of all theoretical mass spectra (SWATH-MS) based quantitative proteomics analysis of differentially expressed proteins (DEPs) at different time points after infiltration of *P. infestans* culture filtrate in *N. benthamiana.* They identified a total of 4401 proteins and 1429 DEPs, of which 6 DEPs were proved to be involved in PTI responses. An additional study on PRR-based resistance in potato is targeting Pep-13/25, which are well-characterized MAMPs in *Phytophthora* species. Lin et al. conducted genetic mapping and characterization of a potato locus conferring perception of Pep-13/Pep-25 present in *P. infestans* and other plant pathogenic *Phytophthora* species. They developed a mapping population using wild potato species and use bulked-segregant RNAseq to map the putative receptor to a 0.081 cM region on the top of chr 3.

Plant viruses also represent limiting factors leading to a loss in the quality and quantity of potato. Due to their limited coding capacity, plant viruses depend on various host factors for successful infection. Loss of function of these factors will result in recessively inherited resistance, and therefore, are also described as recessive resistance or susceptibility genes. Chen et al. discovered the role of a plant eukaryotic translation initiation factor StnCBP as a recessive resistance factor for potato virus S (PVS) by recognizing the viral coat protein.

A major bacterial pathogen is *Ralstonia solanacearum* (Rs), the causal agent of potato bacterial wilt. Type III Secretion System Effectors (T3Es) play a vital role in the interaction. Investigating the T3Es that are recognized by host resistance proteins is an effective method to uncover the resistance mechanism of potato against *R. solanacearum*. Huang et al. employed comparative genomics to identify novel effectors of the Rs strain HA4-1, which is specifically recognized by the wild potato ALB28-1. They found that ectopic expression of two effectors, RipA5 and the newly identified RipBS, in the pathogenic strain HZAU091 of ALB28-1 partially reduced virulence, whereas individual mutations in four unique effectors RipS6, RipO1, RipBS, and Hyp6 in HA4-1 promote virulence.

The soil-borne fungus *Synchytrium endobioticum* is causing potato wart disease. Yan et al. investigated the response of potato cultivar ‘Qingshu9’ against *S. endobioticum* at the transcriptional level, and identified differentially expressed genes (DEGs) in the early and advanced stages of infection. They also identified 17 distinct modules of co-expressed genes using gene co-expression networks.

Altogether, these studies on the diversity of potato pathogens, including oomycetes, virus, bacteria and fungi, promise to contribute to achieving genetic disease resistance in potato.

Abiotic stresses are becoming increasingly important worldwide due to the ongoing global climate change and the increase in agrochemical utilization. Salt stress is a significant threat to potato production in facility cultivation environments, and the mining of salt tolerance genes requires further study. Jing et al. showed that proline homeostasis is critical for potato plantlet growth under salt stress, and identified the TF-hub gene *StGLK014720* as a regulator of two structural genes.

Additionally, environmental factors such as light and temperature influence anthocyanin biosynthesis. The molecular mechanisms regulating anthocyanin biosynthesis were profiled in potato tubers. Zhang et al. studied the gene expression and anthocyanin metabolites of Purple Viking tubers and its red and purple-skin mutants. They discovered that the conversion of certain anthocyanins was blocked in the red tubers and identified a specific gene, *OMT30376*, involved in the transformation of anthocyanins in potato tubers. Zhao et al. analyzed potato RM-210 tubers that turn purple when exposed to light and found that the *StMYBA1* gene was highly correlated with anthocyanin accumulation and could activate the expression of structural genes under light conditions, promoting anthocyanin biosynthesis in potato.

Continuous potato cropping systems cause yield reduction, soil-borne disease aggravation, and soil degradation, but crop rotation can alleviate these negative effects. Qin et al. explored the effects of continuous cropping obstacles on soil biochemical properties and microbial communities in North China. Data from their 4-year field work provided valuable information to improve the management of potato cultivation.

In sum, in this special issue, a diversity of major traits that are essential for a sustainable production of the potato crop are presented. The underlying genes are identified and characterized, and their function in growth and development, tuber nutrition and morphology, abiotic stress, and disease resistance and cropping systems.

## Author contributions

JD prepared the first draft of this editorial. VV and BS revised the editorial. All authors approved the editorial for publication.

